# Virus-Based Immuno-Oncology Models

**DOI:** 10.3390/biomedicines10061441

**Published:** 2022-06-18

**Authors:** Juliana Sitta, Pier Paolo Claudio, Candace M. Howard

**Affiliations:** 1 Department of Radiology, University of Mississippi Medical Center, Jackson, MS 39216, USA; jsitta@umc.edu; 2 Department of BioMolecular Sciences, Department of Radiation Oncology, Cancer Center & Research Institute, University of Mississippi Medical Center, Jackson, MS 39216, USA; pclaudio@umc.edu

**Keywords:** immunotherapeutic, immune-oncology, oncolytic virus, vaccinia virus, humanized mice, cancer, tumor microenvironment, tumor-associated macrophages

## Abstract

Immunotherapy has been extensively explored in recent years with encouraging results in selected types of cancer. Such success aroused interest in the expansion of such indications, requiring a deep understanding of the complex role of the immune system in carcinogenesis. The definition of hot vs. cold tumors and the role of the tumor microenvironment enlightened the once obscure understanding of low response rates of solid tumors to immune check point inhibitors. Although the major scope found in the literature focuses on the T cell modulation, the innate immune system is also a promising oncolytic tool. The unveiling of the tumor immunosuppressive pathways, lead to the development of combined targeted therapies in an attempt to increase immune infiltration capability. Oncolytic viruses have been explored in different scenarios, in combination with various chemotherapeutic drugs and, more recently, with immune check point inhibitors. Moreover, oncolytic viruses may be engineered to express tumor specific pro-inflammatory cytokines, antibodies, and antigens to enhance immunologic response or block immunosuppressive mechanisms. Development of preclinical models capable to replicate the human immunologic response is one of the major challenges faced by these studies. A thorough understanding of immunotherapy and oncolytic viruses’ mechanics is paramount to develop reliable preclinical models with higher chances of successful clinical therapy application. Thus, in this article, we review current concepts in cancer immunotherapy including the inherent and synthetic mechanisms of immunologic enhancement utilizing oncolytic viruses, immune targeting, and available preclinical animal models, their advantages, and limitations.

## 1. Introduction

Immunotherapeutic targets have emerged in the past decade as a promising addition to the oncology treatment arsenal for selected types of cancers [1,2]. With the rapid development of new immune anticancer drugs and viruses, accurate and reliable preclinical validation including assessment of tumor growth and response, evaluation of complications, drug resistance, and mechanistic effects became of utmost importance to expedited drug development [3]. Recent advances, including the recognition by the Nobel Prize in 2018 of Dr. James P. Allison and Dr. Tasuku Honjo for their discoveries in immune-oncology treatment, highlight the current research focus on the development of new immunotherapeutic drugs [3,4].

With the advent of new immunotherapeutic targets and new developments in oncolytic virus (OV) immunotherapeutic applications, problems with preclinical validation and safety have emerged [5]. Classically, simple preclinical models often use orthotopic or syngeneic tumors, either in immunocompromised or immunocompetent mice [6]. Although they are essential to initial target and mechanism determination as well as time and cost-effective, these models have demonstrated limited reproducibility in clinical trials [7].

Along those lines, multiple animal models have been developed in an attempt to suffice the need for a reliable preclinical assessment of these drugs. Humanized mice, which comprise immune-deficient mice engrafted with human hematopoietic cells and therefore with a human immune system, are the most promising models [8]. Each of the humanized mice models has advantages and disadvantages that constrain their use with certain targets. These characteristics and models will be reviewed in this article as well as their application in current and past drug therapies.

## 2. Oncologic Immunomodulation

The relationship between tumor tissues and the immunologic system has been increasingly explored with a new concept of immunologic modulation in the fight against cancer cells. The thought that tumors escape immune surveillance is not recent, but evolved significantly in the past decade with the development of immune checkpoint inhibitors (ICI), specifically the ones targeting the cytotoxic T lymphocyte antigen 4 (CTLA4) and programmed cell death 1 (PD1), that showed encouraging results in initial clinical trials [9,10]. Ipilimumab and tremelimumab emerged as therapeutic strategies to augment anti-tumor immunity against cancer [11]. Although initial studies demonstrated improved outcomes with a combination of ICI therapy, long-term follow-up showed no significant clinical difference of combined nivolumab and ipilimumab survival compared to single therapies [12]. Furthermore, ICI’s high cost and immune-related adverse effects became a concern [13,14]. Thus, there is growing interest in complementary agents with safer toxicity profile.

CTLA4 intrinsic function is closely linked to T lymphocyte modulation and a major responsibility for immunologic tolerance [15]. CTLA4 is released from intracellular vesicles to the immunologic synapse following T cell receptor (TCR) activation [16]. CTLA4 attenuates T cells by competitive binding to ligands B7-1 and B7-2. CD28 interaction with ligands B7-1 and B7-2 is intrinsically positive through downstream signaling mediated by phosphoinositide 3-kinase (PIK3) and protein kinase B (AKT) [17,18]. Both co-stimulatory positive and negative interactions are similar in strength, allowing for modulation swift. CTLA4 acts primarily in sites of lymphocyte priming but may also be encountered in varying degrees in peripheric tissues where antigen-presenting cells and activated lymphocytes express B7 ligands [19].

In addition to intrinsic function, CTLA4 also modulates T lymphocyte activation extrinsic mechanisms through regulatory T cells (Tregs) modulation. Loss of extrinsic CTLA4 modulation is sufficient to induce aberrant T cell activation and autoimmunity. In this context, CTLA4 appears to modulate B7 ligand activation in antigen-presenting cells, possibly by a mechanism of trans-endocytosis [20]. CTLA4 modulation occurs by selective competitive attenuation of high strength TCR expression, allowing for medium-strength T cell activation [21].

The PD1-PD-ligand 1 (PD-L1) pathway has shown to significantly improve survival in preclinical and clinical trials and is currently in clinical use for many types of cancer. PD-L1 is expressed by many types of cells including tumor cells while PD-L2 is expressed mainly by normal dendritic cells [22]. Thus, the PD-1 mechanism primarily acts in the periphery. PD-1 signaling pathway is activated upon T and B cell activation and, different from CTLA4, appears to directly inhibit TCR activation through activation of the downstream signaling mediated by the tyrosine phosphatase SHP2 [23,24]. In addition, CD28 has also been demonstrated to confer a primary target for PD-1, indicating a common pathway with CTLA4. The PD-1—PD-L1 interaction attenuates T lymphocyte immune response, and in the setting of cancer, is a major contributor to impaired anti-cancer immune response [25].

**Figure 1:**
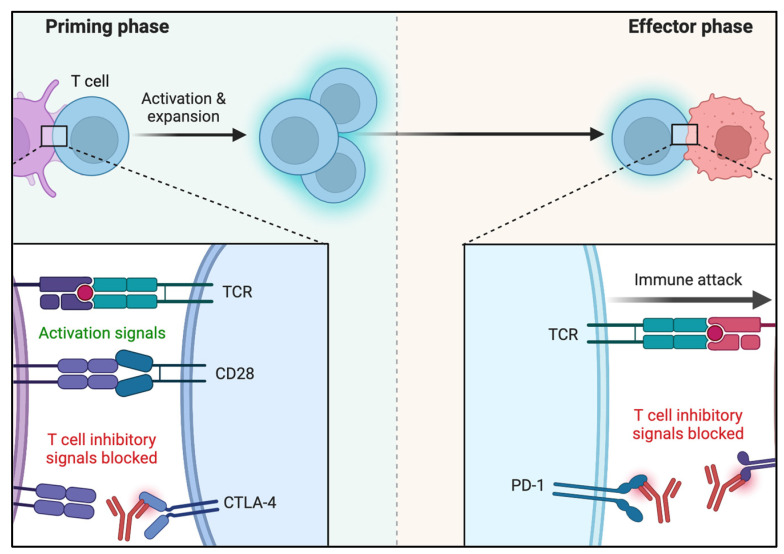
Molecular mechanisms of CTLA-4 and PD-1 inhibitory T-cell activation. Reprinted from “Blockade of CTLA-4 or PD-1 Signaling in Tumor Immunotherapy”, by BioRender.com (2022) (accessed on 6 March 2022). Retrieved from https://app.biorender.com/biorender-templates (accessed on 6 March 2022).

Research has shown that the higher the mutation rate of a tumor, the more likely it is to produce neoepitopes, cell surface biomarkers, and, thus, to elicit immune response [26]. The cancer–immunity interaction complex, however, is not at all simplistic. Besides tumor cell heterogeneity, patients’ background immune capabilities influence tumor immune response and depend on multiple poorly measurable epigenetic variables. The presence of chronic antigen cell activation has been demonstrated to induce T-cell exhaustion [27]. Long-term PD-1 activation induces a cell energy switch by attenuating glycolysis and increasing fatty acid metabolism [28]. CTLA4 attenuates T-cell glycolysis but without a definite lipid metabolism effect [29]. Along those lines, chronic virus infection and cancer model systems have demonstrated that once antigen-specific T-cell reaches exhaustion, this heritable pattern of response is maintained even with reduced antigen presentation [30]. Epigenetic changes have been demonstrated to promote immune check point and T cell exhaustion genes possibly by inducing their upregulation which ultimately may hamper the immunotherapeutic rescue in cancer patients [31,32]. Figure 1 summarizes the main primary and peripheral effects of PD-1 and CTLA-4.

One of the key components in tumor microenvironment (TME) immunosuppression is the tumor-associated macrophages (TMA). TMAs have been recognized as one of the main contributors to ICI response failure in solid tumors [33,34,35]. Typically, macrophages may display immunosuppressive, immunogenic, or both functions in varying degrees within the TME. However, researchers have demonstrated predominantly immunosuppressive pro-tumor TMA activity, particularly in poorly ICI responsive tumors [33]. Moreover, higher TMA density correlated with an increased likelihood of advanced tumors, poor prognosis, and higher ICI resistance [36]. Consequently, TMA recently became a new therapeutic target, with emerging research exploring TMA modulators to enhance ICI response [37].

Macrophage activation is typically characterized by two polarizations: The M1, which is pro-inflammatory; and the M2, which is immunosuppressive. M1 macrophages are classically activated by interferon-gamma (IFNγ) and lipopolysaccharides and present anti-tumor activity induced by nitric oxide synthase and release of cytotoxic reactive oxygen species and pro-inflammatory cytokines [38,39]. Within the innate immune system arsenal, activated M1 macrophages attract natural killer (NK) cells and dendritic cells (DCs) through the expression of various chemokines including chemokine ligand 20 (CCL20), C-X-C motif chemokine ligand 10 (CXCL10), and C-X-C motif chemokine ligand 11 (CXCL11) [40]. These same chemokines are also responsible for the activation and recruitment of T cells, and, thus, influence the tumor’s tumor infiltrating lymphocytes (TIL). NKs and DCs are then activated by interferon-alpha (IFNα) and interleukin-12 (IL-12). NKs and DCs, in turn, activate macrophages by secreting IFNγ, IL-12, and interleukin-15 (IL5), which are pro-inflammatory chemokines [41].

Macrophages activated by interleukin-4 (IL-4), the M2 subtype, are called tumorigenic given their ultimate ability to express growth-promoting, pro-angiogenic, and extracellular matrix remodeling signals via vascular endothelial growth factor, interleukin-8 (IL-8), matrix metalloproteinase, transforming growth factor β and T cell suppression molecules [42,43,44]. CD169(+) macrophages as lymph node-resident antigen presenting cells (APCs) dominate early activation of tumor antigen-specific CD8(+) T cells and have been demonstrated to play a crucial role in tumor immune activation [45,46].

NK cells, another component of the innate immune system, contribute to oncolysis by phagocytosis and cytotoxic mechanisms and are APC-independent [47]. NK activation is determined by stress-related molecules such as NKp46, NKG2D, 2B4, CD2, and DNAM. NK activation leads to granzyme and perforin-mediated cytolysis as well as Fas-ligand-induced tumor apoptosis. NK cells have been also found to play an important role in the adaptive immune response through DC and M1 macrophage activation [48,49]. NK density in the tumor tissue has been demonstrated to indirectly correlate with tumor growth and metastases, independent of monocyte/macrophage gene-specific expression [50]. This axis, however, may be impaired by tumor-derived prostaglandin E_2_, which both decreases NK cell function and causes downregulation of the XCR1 and CCR5 chemokine receptors on DCs [49].

### Mechanisms of Tumor Evasion

Although ICI therapy has demonstrated promising results in cancer treatment, response in solid tumors remains limited. Innate and acquired resistance to immunotherapy are known limiting factors [51]. Understanding the mechanisms involved in the immunologic pathways within the tumor microenvironment is crucial for improved targeted therapy development. Several authors have previously described the cancer–immune system interaction in stages that range from elimination, equilibrium, and escape [52]. 

The first step in immunologic response consists of the capture of tumor antigen from the tumor microenvironment by APCs such as dendritic cells [53]. Once APCs reach lymph nodes, antigens are presented to naïve T-cells, which subsequently activate and mature effector T cells. The effector T cells are then able to recognize and eliminate tumor cells presenting such antigen [54]. When immunogenic response is active, the so-called elimination and equilibrium phases take place and tumor growth is damped or controlled. However, once the tumor cell selection process overcomes immunogenic response capabilities, the tumor is able to “escape” immunogenic elimination and TILs are dramatically reduced [55].

Since an adequate immunologic response is dependent on the optimal function of multiple variables, any defect could generate impaired immune elimination of tumor cells. For instance, tumor cells with a low mutational burden are thought to produce fewer neoantigens and, thus, are less responsive to immunotherapy [56,57]. Furthermore, only neoantigens that are recognizable by the activated T cell will result in immunologic elimination. Of note, not all tumor cell mutations generate immunogenic neoantigen. Frameshift mutations are typically more likely to generate immunogenic neoantigens [58]. A different approach also demonstrated that neoantigens that closely resemble pathogen antigens demonstrate increased immunogenicity [59,60].

The mismatch repair (MMR) is an example of immunologic response biomarker that gained importance for pembrolizumab recommendation guidelines. MMR is the process responsible to correct for errors generated during DNA replication. MMR deficient tumors demonstrated hypermutational profile with strong neoantigen production that is highly immunogenic upon PD-L1 pathway inhibition. However, the neoantigen pattern of expression and anti-tumor immune response is unpredictable; thus, not all tumors will respond to PD-L1 pathway inhibition [61]. This was the rationale for the first ICI approval based on biomarker expression rather than pathologic subtype [62].

Additional mechanisms contributing to a decreased tumor immunogenic response include poor lymphocyte infiltration, resistant DC phenotypes, an abundance of Tregs within the tumor, which are immunosuppressive and hamper priming of naïve T cells, and recruitment of effector T cells.

**Figure 2:**
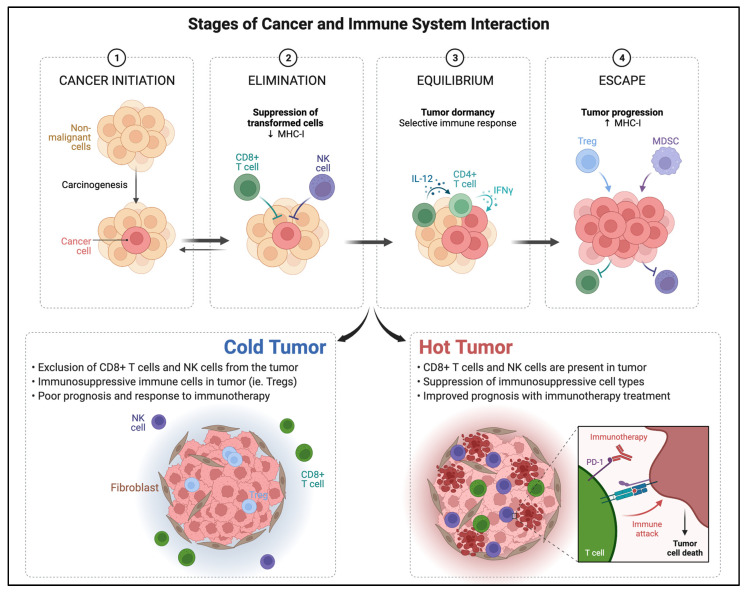
Outline of the stages of cancer and immune system interaction and tumor phenotypes classified by immunogenicity within the tumor microenvironment at the cellular level. Adapted from “Cancer Immunoediting” and “Cold vs. Hot tumors”, by BioRender.com (2022) (accessed on 28 March 2022). Retrieved from https://app.biorender.com/biorender-templates (accessed on 28 March 2022).

Based on intrinsic immunologic characteristics of the TME, a classification of tumor phenotypes based on their immune activity has been proposed (Figure 2). The first phenotype is the immune inflamed or hot tumor. In this profile, there are abundant CD4+ and CD8+ cells, effector T cells are near tumor cells, and there is an abundance of cytokines and proinflammatory markers [63]. In the hot tumor phenotype, PD-L1 is upregulated by pro-inflammatory cytokines including IFN-γ from activated T cells. In the cold-tumor immunologic phenotype, however, PD-L1 expression is downregulated and innate immune response is ineffective, a characteristic oncogenic pathway intrinsic to the tumor. Cold tumors present higher immunosuppressive cell populations such as Tregs, TAMs, and myeloid-derived suppressor cells [64]. Hot tumors are characteristically more responsive to PD-L1 checkpoint inhibitors and immunotherapy compared to cold tumors but may progress to exhaustion.

## 3. Oncolytic Viruses and Immuno-Oncologic Modulation

OVs present a natural or genetically engineered tropism for tumor cells that can be further enhanced to increase both innate and adaptive immune responses. Over the years, OVs, particularly adenovirus, have been continuously modified to increase tumor selectivity and minimize toxicity. OV mechanism of action is thought to occur through three main mechanisms: the primary lysis of tumor cells caused by intracellular viral replication, gene modification delivery, and the secondary increase in antigen-presenting molecules leading to an increased adaptive immunologic response. One of the main advantages is that given the selective nature of OVs, these mechanisms may be used systemically to reach tumor metastatic tissue that was not directly inoculated.

Viruses and other pathogens naturally stimulate stronger immune responses than over-expressed self-antigens normally encountered in solid tumors. OVs have evolved through the years to express multiple cell receptors and lack intrinsic replication capabilities. OVs mechanism rely on virus-mediated cytolysis promoting the release of pathogen-associated molecular patterns (PAMPs) and danger-associated molecular patterns (DAMPs), which are cancer cell death sensors [65]. Tumor oncolysis and release of PAMPs and DAMPs, which are recognized by pattern recognition receptors (PRR), lead to DC activation and subsequent CD4 and CD8 T cell priming.

**Figure 3:**
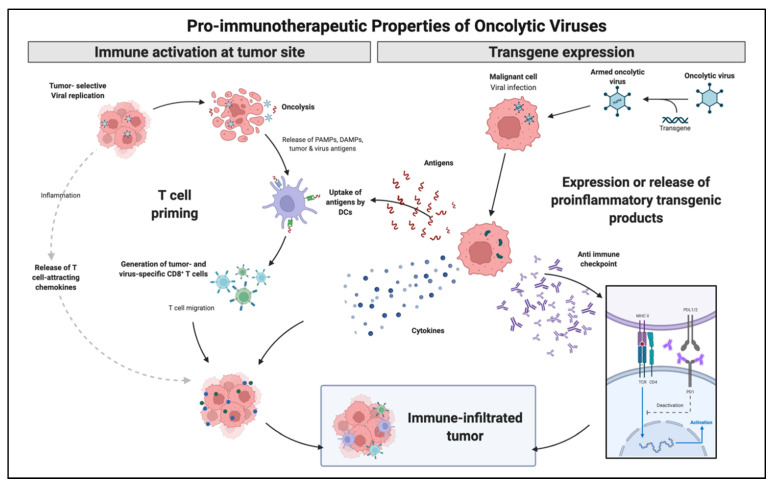
Immune enhancement mechanisms explored utilizing OVs strategies. OVs may generate increased immunologic response by direct cell lysis with antigen release and local inflammation as well as artificially generate pro inflammatory molecules by transgene expression. Adapted from “Properties of Oncolytic Viruses”, by BioRender.com (2022) (accessed on 28 March 2022). Retrieved from https://app.biorender.com/biorender-templates (accessed on 28 March 2022).

With a growing body of evidence in immunologic modulation for cancer therapy, OVs followed a similar trend by genetically adding immunologic capabilities. Talimogene laherparepvec (T-VEC) is a herpes-simplex virus encoding granulocyte-macrophage colony-stimulating factor (GM-CSF) and was the first OV-based immunotherapy to reach a phase III trial. It is currently FDA approved for the treatment of selected patients with metastatic melanoma that are not candidates for surgical resection. Following intratumoral administration, T-VEC induces cell lysis followed by the release of tumor antigen and subsequent circulation of tumor-specific effective T-cells. Thus, although the administration is local, the treatment effect reaches systemic levels. GM-CSF controls both myeloid differentiation and the function of mature presenting antigen cells. This effect has been replicated with adenovirus (Ad5-D24-GMCSF), which demonstrated complete tumor regression and tumor-specific cytotoxic T lymphocyte response [66]. Figure 3 illustrates OVs immune modulation mechanisms.

### Oncolytic Viruses and Immunotherapeutic Drugs

Sipuleucel-T autologous cell-based immunotherapy vaccine FDA-approved for the treatment of hormone-refractory prostate cancer. Production is individualized by leukapheresis of the patient’s peripheral blood antigen-presenting cells followed by ex vivo antigen loading with prostate acid phosphatase (PAP) and GM-CSF enhancement [67]. Approval was based upon the IMPACT trial, a multicenter phase 3 clinical trial comparing sipuleucel-T or placebo in men with asymptomatic metastatic castration-resistant prostate cancer. In this population, sipuleucel-T showed modest improvement in overall survival (25.8 months in the sipuleucel-T group vs. 21.7 months in the placebo group) but no effect in time to disease progression [68]. Based on the effects of cancer vaccines such as Sipuleucel-T, several OVs armed with GM-CSF (HSV T-Vec, VV JX-594, Ad Ad5/3-D24-GMCSF, and CG0070 have entered clinical trials [69].

The combination of immunotherapy drugs has been attempted as an approach to increase tumor cell response. However, the combination of immunotherapy drugs carries concerns with increased adverse effects [70]. The concept of utilizing a combination of oncolytic virus and immunotherapy emerged to enhance immunomodulation, particularly to overcome immunosuppressive effects within the TME while maintaining a safety profile. Along those lines, a recent phase II clinical trial demonstrated the improved response of T-VEC combined with ipilimumab with decreased visceral lesion decreases in 52% of patients in the combination arm and 23% of patients in the ipilimumab arm. This study included selected patients bearing advanced melanoma. There was no significant increase in adverse events with the combination approach [71].

With the same concept of vaccine cancer therapy, authors recently reported on the use of cancer stem cells lysate-pulsed dendritic cell vaccine with induced tumor-specific humoral and cellular immunologic response [72,73,74]. Cancer stem cells are encountered in higher number in selected tumors subtypes and are known for their high differentiation capabilities, contributing to tumor heterogeneity and therapy resistance [75]. Although innovative, response with single cancer stem cell vaccine therapy was limited by the immunosuppressive tumor microenvironment that hampered immunologic response [76]. In a more recent article, authors explored the use of combination dual or triple therapy with PD-L1 or CTLA4 inhibitors, demonstrating increased T cell proliferation, improved tumor-specific CD8 + T cell response, and inhibition of tumor necrosis factor-beta (TNFβ), resulting in a dramatic tumor response in animal models [77].

Chimeric antigen receptor-modified T cell therapy (CAR-T) is an adoptive autologous T cell therapy strategy targeting cells or TME. CAR-T synthetically generates personalized effector T cells with a high affinity for tumor antigens independent of MHC [78]. CAR-T therapy demonstrated dramatic clinical response in B cell malignancies [79,80,81,82]. However, CAR-T therapy in solid tumors is much less successful, likely secondary to immunosuppressive TME changes [83,84,85]. Li et al. evaluated the combination of CAR-T therapy and oncolytic adenovirus expressing TNFβ receptor II-Fc (rAd.sT) in a triple-negative breast cancer model [86]. This modified adenovirus targets and inhibits TNFβ signaling, decreasing immunosuppressive effects in the TME [87]. The authors constructed CAR-T cells targeting mesothelin, a protein that is normally expressed by various mesothelial tissues but also over-expressed by a wide range of cancers [88]. The authors demonstrated moderate mesothelin expression in the MDA-MB-231 cell line. The combination therapy elicited increased apoptosis and the author detected a synergistic effect in the expression of IL-2 and IL-6 cytokines, important immunogenic cytokines.

In a different work by Watanabe et al., an engineered oncolytic adenovirus expressing tumor necrosis-alpha (TNFα) and interleukin-2 (IL2) (Ad5/3-E2F-D24-TNFa-IRES-IL2 or Ad-mTNFa-mIL2) was administered to humanized mice bearing pancreatic adenocarcinoma xenografts. Pancreatic adenocarcinoma has a highly immunosuppressive TME. The authors demonstrated the increased intensity of T cell infiltration with sustained tumor regression after combined meso-CAR-T cells and oncolytic virus therapy. Pancreatic adenocarcinoma was resistant to meso-CAR-T cells alone, but co-administration with Ad-mTNFa-mIL2 elicited tumor regression. Moreover, Ad-mTNFa-mIL2 demonstrated induced increased M1 polarization of macrophages and DC maturation compared to control adenovirus, again indicating that oncolytic therapy enhances innate and adaptive immunity [89].

TMAs and OV interplay, however, is not completely clear and often contradictory in the literature. Although it has been reported that tumor inflammation inhibits viral replication through interferon release, some tumors have demonstrated a response to virus-based anti-tumor immune activation [90,91]. This relationship is, however, not linear and the type of macrophage polarization (M1 or M2) appears to vary by tumor and type of virus [92]. Recently, authors tested the anti-tumor effect of a recombinant Newcastle disease virus (MEDI5395) expressing GM-CSF and demonstrated enhanced immune-cell activation and pro-inflammatory cytokine release in vitro [93].

## 4. Delivery Systems

Neutralizing anti-viral antibodies have been identified as one of the main obstacles to oncolytic virus tumor response. Neutralizing antibodies may originate from the previous contact with a similar community-based virus or can be generated upon contact with the inoculated virus, thus, ultimately leading to resistance to oncolytic therapy. A multitude of materials and delivery systems have been developed to increase OVs delivery efficiency and have been previously reviewed elsewhere [94]. One recent advancement that allowed for overcoming these limitations, has been the development of a systemic site-specific delivery system where ultrasound (US) contrast agents, microbubbles (MBs), are used as delivery vehicles for adenoviruses (hAds). The hAds loaded inside shells of acoustically active, lyophilized, lipid-encapsulated, perfluorocarbon-filled MBs, are released when MBs are destroyed by US at the tumor site. These bubbles range between 2.5 and 4.5 µm, and after injection into the bloodstream, they can re-circulate through the vascular system numerous times for several minutes with minimal accumulation and interaction [95,96,97,98,99]. Using the MB gene transfer system, we have selectively transferred therapeutic genes into tumors in immune-deficient mice [95,98].

Additionally, using the MB gene transfer system in healthy immunocompetent C57BL/6 mice or mice bearing a syngeneic TRAMP-C2 prostate tumor, we protected systemically delivered adenoviral vectors from the innate and adaptive immune system preventing the off-target distribution of the viruses [100].

**Figure 4:**
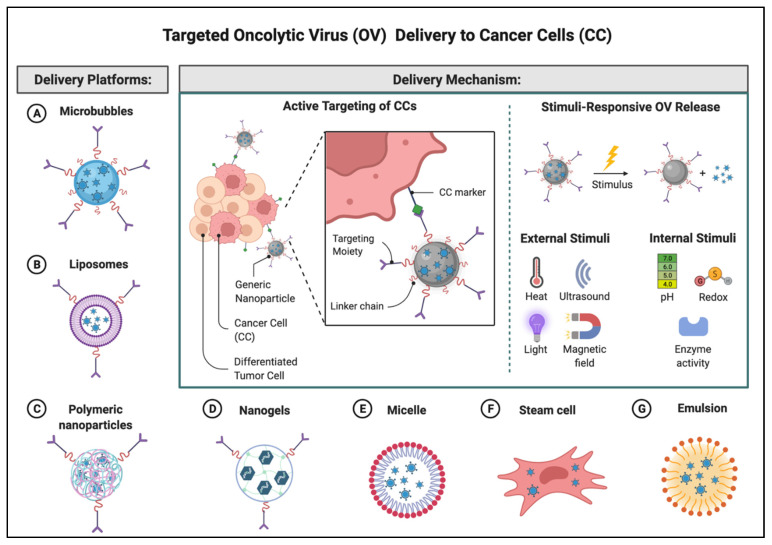
Targeted OV delivery to cancer cells. This illustration highlights a non-exhaustive list of the delivery platforms that have been reported in the literature to protect OVs from neutralizing antibodies as well as enhance targeted delivery by external or internal stimuli. Many are carriers but also facilitators and may be either organic or inorganic in nature. Adapted from “Nanoparticle-Mediated Targeted Drug Delivery to Cancer Stem Cells”, by BioRender.com (2022) (accessed on 28 March 2022). Retrieved from https://app.biorender.com/biorender-templates (accessed on 28 March 2022).

In sum, extensive research has demonstrated successful oncolytic virus encapsulation and neutralizing antibody escape by utilizing micro and nanoparticles. Recently, a new approach has been described using mesenchymal stem cells (MSC) carriers, known as trojan horses, which are inoculated ex vivo with oncolytic viruses. MSCs allow for antibody neutralization shielding, tumor penetration, and the advantage of intracellular replication [101] Figure 4 illustrates several potential delivery platforms currently being explored for targeted OV delivery.

## 5. Preclinical Models

Rodents have been historically the most common animals to star in preclinical cancer research. Over the years, the constant development of new rodent models, particularly mice, have allowed an improved understanding of human tumor biology, pathophysiology, and anticancer drug response. Choosing an appropriate preclinical model, however, is not as simplistic, and directly influences the predictive validity of the study. Typically, to advance from a murine to a human model of cancer research, mice models must lack immunity to avoid innate cancer cell rejection. Thus, with the advent of oncolytic virus cancer immunotherapeutic, the common mice models became an important limitation for preclinical validation. There was a need for a model that would mimic or more closely reproduce the human immunologic response to the studied pathology or drug. This need led to the development of mice xenografted with human immune and cancer cells.

Humanized mouse models are primarily made from immunodeficient mice engrafted with human-derived cancer cells, while also bearing human-derived immune cells.

The first humanized mice model described in the literature used mice bearing severe combined immunodeficiency disease (SCID) determined by a mutation in the protein kinase DNA–activated catalytic polypeptide [102]. These mice had a deficiency of lymphocytes T and B, which allowed for modest engraftment of heterologous cells. Further incorporation of Rag mutations impaired murine adaptive immunity by disrupting the V(D)J (variable-diversity-joining) recombination [103]. The SCID mutation was then backcrossed with non-obese diabetic (NOD) mice further decreasing murine innate and adaptive immunity [104].

The next generation of humanized mice included a knock-out of interleukin-2 receptor subunit gamma (Il2rg) that is responsible for the development of natural killer cells in NOD-SCID mice. Consequently, these additional IL2 mutations generated a mouse not only deficient in lymphocytes B and T, but also bearing defective NK cells, impaired macrophage function, complement activity, and DC function. From this model, the two most popular immunodeficient mouse models were created: The NOG mouse, which included a NOD mouse with mutated Il2rg leading to truncation of the intracellular signaling pathway; and the NSG mouse, which comprise a complete null Il2rg mutation [105].

### Human Immune System Engraftment Approaches

Humanized mice may be engrafted with different approaches, each delivering varying degrees of immunologic response. Here, we summarize the concepts of each approach.

The simplest approach consists of the inoculation of human peripheral blood mononuclear cells to immune-deficient mice, known as the peripheral blood leukocyte-humanized immune system (PBL-HIS) model. Human reconstitution is obtained relatively fast ranging between 3–5 days, allowing for a robust activated human T cell lineage. The main disadvantage is the rapid development of graft-versus-host disease that inevitably takes place in 4–6 weeks. This timeline limits applicability to long-term studies [106,107]. Thus, this model is mainly useful for short-term studies focused on graft versus host disease and T cell targets, since it also lacks human myeloid components, platelets, and red blood cells.

A more complex approach was subsequently developed to partially overcome the myeloid lineage limitation of PBL-HIS models by engrafting umbilical cord blood, adult bone marrow, mobilized peripheral blood cells, or fetal liver, known as hematopoietic stem cell-HIS (HSC-HIS). To avoid the early development of graft-versus-host disease, these mice are subjected to a sub-lethal gamma radiation dose before engraftment. This model allows reconstitution of functional multilineage human hematopoietic cells and longer over life [108]. Time to reconstitution is typically longer, ranging between 3–4 weeks [109,110,111]. This model’s main limitations consist in the inevitable mouse cytokine and growth factors interactions that are not as effective as the syngeneic kind, as well as the dysfunctional activation of T cells by human antigen-presenting cells since these cells are in contact only to mouse major histocompatibility complex (MHC). Additional disadvantages include the complexity of engraftment, relatively higher cost, time to reconstitution, and limited availability of engraftment tissues. This improved model is suitable for long-term studies investigating hematopoiesis, immune checkpoint inhibitors, oncolytic virus, cell-mediated immunity, and adoptive cell therapy [112].

The bone marrow–liver–thymus (BLT) model is a higher complexity mouse model that uses fetal liver, fetal thymus, and autologous fetal liver hematopoietic stem cells allowing for a complete human immune system reconstitution. Mice in this model also require sublethal gamma irradiation before engraftment with delayed graft versus host disease development. Disadvantages are similar to HSC-HIS models, with higher relative costs, the complexity of human engraftment, time to reconstitution, and availability constraints of tissues [113]. These complex models have been used in immunotherapeutic and vaccination applications.

Humanized mouse models have significantly advanced research of viral infections throughout recent years. The study of oncolytic virus as immunotherapeutic agents presents particular challenges to preclinical model development due to specific immune cell functionality that needs to be assessed to reliably evaluate tumor response, all while accepting human tumor cell engraftment. As mentioned earlier, humanized mice bearing lymphoid tissue are the most commonly available, simplest approach, and valuable tool for OV and adaptive immunity research.

OVs targeting innate immunity and the TMA have more limited preclinical model availability. To replicate the human myeloid cell maturation, the animal must bear not only human cells but also the stimulatory factors responsible for myeloid cell maturation. Mice strains have been developed for this objective by including human cytokines knock-in including human stimulating factors such as IL-3, IL-15, GM-CSF, and macrophage colony-stimulating factor [114]. The additional incorporation of Rag2/Il2rg allowed for more physiological levels of NK cells [107]. Human innate immune cell functionality in mice is still very limited and further improvement is needed [107,114].

## 6. Conclusions and Future Perspectives

OV’s immune-oncology applications hold great promise, particularly in combination with ICIs to improve solid tumor response. Although ICIs demonstrate a significant tumor response in hematologic malignancies, the response is limited in solid tumors. OVs have been recently explored to target or express pro-inflammatory cytokines and molecules to enhance immune cell transfection within solid tumors with promising results.

Many challenges need to be overcome to improve the clinical application of OVs therapy either as single or combined treatment. Clinical safety related to the genetically engineered virus depend on many factors involving the gene expression stability, large scale production and amplification, targeted delivery, evasion from neutralizing antibodies, appropriate preclinical safety evaluation, and tumor response assessment. Progressive development of OVs over the years demonstrated its multimodal functionality such as cell lysis, immunomodulation, and gene therapy. Oncologic OV’s applications have demonstrated over the years better results with combined therapy, which is likely secondary to the tumor’s heterogeneity. Thus, future clinical applications are more likely to involve multiple therapeutic modalities.

This article summarized important concepts in the development of virus-based immune-oncology therapy development.
